# Behavioral pharmacology of novel synthetic indazole, indole, and benzimidazole cannabinoids in rodents

**DOI:** 10.1186/s42238-026-00390-3

**Published:** 2026-01-13

**Authors:** Ritu A. Shetty, Rebecca D. Hill, Jared A. McDonald, Nathalie Sumien, Michael J. Forster, Michael B. Gatch

**Affiliations:** Department of Pharmacology and Neuroscience, Center for Neuroscience Discovery, University of North Texas Health Science Center, 3500 Camp Bowie Blvd, Fort Worth, TX 76107-2699 USA

**Keywords:** Abuse liability, Synthetic cannabinoids, Delta-9-tetrahydrocannabinol, Locomotor activity, Drug discrimination, Rodents

## Abstract

**Background:**

Synthetic cannabinoids (SC) are created as alternatives to delta-9-tetrahydrocannabinol (∆^9^-THC) and can cause serious side effects like hallucinations and death. The DEA has identified eleven concerning synthetic cannabinoids: ADB-BUTINACA, ADB-HEXINACA, ADB-4en-PINACA, ADB-FUBIATA, 4F-MDMB-BICA, 4F-ABUTINACA, FUB-144, FUB-AKB-48, 5F-EMB-PICA, 5Cl-AKB-48, and MDA-19. These compounds were tested in rodent models to evaluate their effects on locomotion, discrimination, and potency relative to ∆^9^-THC.

**Methods:**

The eleven SC were tested for locomotor activity in Swiss-Webster mice and drug discrimination (DD) in Sprague–Dawley rats trained to discriminate ∆^9^ THC.

**Results:**

In tests for locomotor depression, all the test compounds were more potent than ∆^9^-THC (ED_50_ = 3.3 mg/kg) except for FUB-144, 5Cl-AKB-48, and ADB-FUBIATA (ED_50_ > 6.1 mg/kg), which produced weak locomotor depressant effects. Similarly, in the DD assay, most compounds substituted fully with greater potency t than ∆^9^ -THC (ED_50_ = 0.55 mg/kg) except for FUB-144 (ED_50_ = 1.44 mg/kg), and 5Cl-AKB-48 (ED_50_ = 3.21 mg/kg). ADB-FUBIATA and MDA-19 tested in doses up to 100 mg/kg, failed to fully substitute for the discriminative stimulus of ∆^9^-THC.

**Conclusion:**

In summary, slight structural differences led to shifts in behavioral pharmacology outcomes in rodent models, which helped predict their potency relative to ∆^9^-THC. Therefore, future research on emerging SCs, along with structure–activity relationships at the receptor level, should include behavioral pharmacology testing of SCs to better predict their abuse potential and toxicity.

**Support:**

Supported by NIDA contract N01DA-18–8936; N01DA-23–8936.

## Background

In 1970, the Drug Enforcement Administration (DEA) classified Δ^9^-THC as a Schedule 1 compound, and therefore, it is illegal to prescribe or possess. This led to the marketing of quasi-legal synthetic cannabinoids (cannabinoid agonists or ‘spice’). First introduced to the market in 2004, these substances became popular starting in 2008 (Spaderna et al. 2013; ElSohly et al. 2019). To classify this ever increasing family of SCs, the EMCDDA introduced a generic model system that includes four components: the head, linker, core, and tail (EMCDDA 2017).

**Fig. 1:**
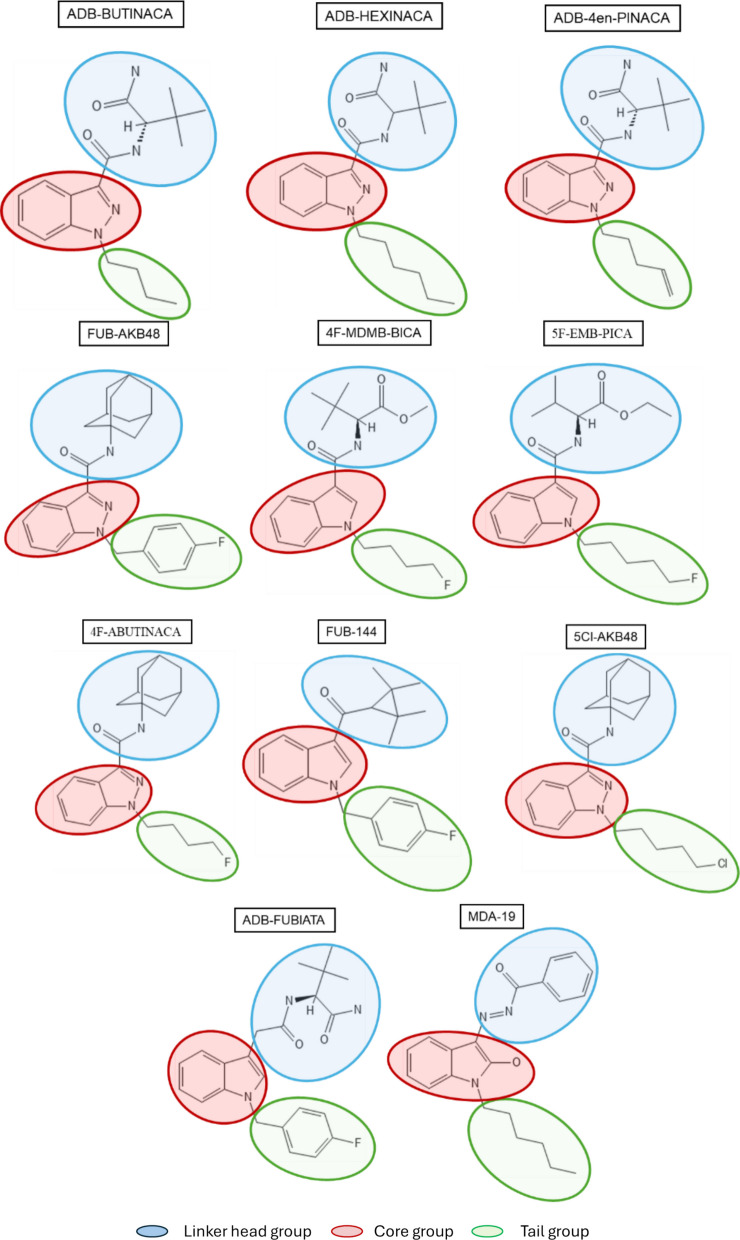
Chemical structures of test compounds. The illustrations depict the chemical structures of the eleven synthetic cannabinoids evaluated. Each structure indicates the linker head (blue), core (red), and tail groups (green)

Due to the increasing prevalence of ADB-BUTINACA, ADB-HEXINACA, ADB-4en-PINACA, ADB-FUBIATA, 4F-MDMB-BICA (4F-MDMB-BUTICA), 4F-ABUTINACA, FUB-144 (FUB-UR-144), FUB-AKB-48 (AFUBINACA, FUB-APINACA), 5F-EMB-PICA (EMB 2201), 5Cl-AKB-48 (5-Cl-APINACA), and MDA-19 (BZO-HEXOXIZID), and the societal impact of these SC, the DEA has identified these compounds as potentially hazardous. These eleven compounds belong to either indazole/indole-3-carboxamide, indole, or oxizid class of synthetic cannabinoid (Shevyrin and Morzherin 2015; Oliveira et al. 2023) as shown in Table 1. Chemical structures with the head, linker, core, and tail components marked are shown in Fig. 1.

**Table 1:**
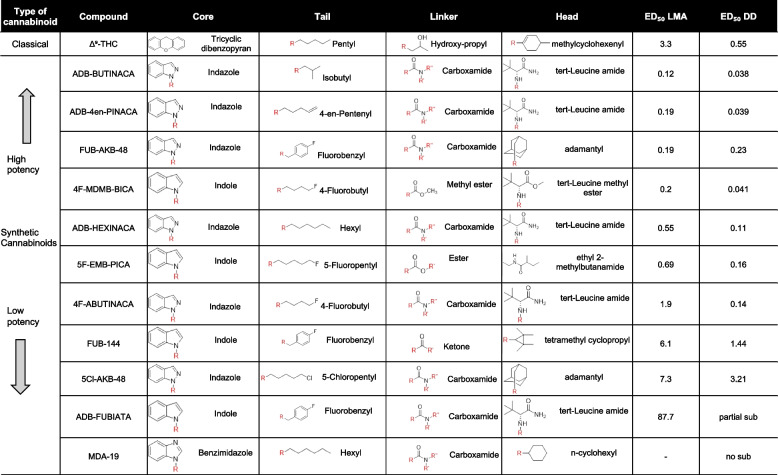
Structures and potencies of synthetic cannabinoids tested

Among the indole-3-carboxamide SC, ADB-BUTINACA was first reported in forensic toxicology in Sweden in 2019 (EMCDDA 2020) and then quickly gained popularity. By 2021, it was detected infused in seized papers from Scottish prisons (Norman et al. 2021). Multiple metabolites of ADB-BUTINCA were detected in urine, blood, kidney and liver. The parent compound was detected in blood collected from patients of suspected drug intoxication, and in postmortem tissues (Kavanagh et al. 2022). Additionally, levels in blood was lower than in liver or kidney in postmortem samples, suggesting a rapid metabolism of this drug and making it challenging to detect (Kavanagh et al. 2022). ADB-BUTINACA has also been identified as part of ‘La Chimique’, a drug mixture consumed in the Mayotte Islands (Richeval et al. 2024).

ADB-HEXINACA, which falls under indazole-3-carboximide SC, was also detected infused in papers seized from Scottish prisons. Although it is less prevalent than ADB-BUTINACA, it has a potency comparable to MDMB-4en-PINACA and warrants monitoring (Kronstrand et al. 2022). A transnational study explored the development of synthetic cannabinoid receptor agonists (SCRAs) across Germany, the United Kingdom, and the United States. Data obtained from 4,427 individuals identified MDMB-4en-PINACA and 4F-MDMB-BINACA as dominant indazole-3-carboxamide compounds. They also identified 5F-EMB-PICA as an indole-3-carboxamide SC (Norman et al. 2021).

In Hungary, 4F-MDMB-BINACA made the headlines after it was identified in 51 police raids and was linked to 11 fatalities (Police News and Information Related 4F-MDMB-BICA Police n.d.). In vitro studies showed that its effectiveness at the CB1 receptor was 7.4 times greater than Δ^9^-THC, with maximum values reported at 179% and 253% (Dvoracsko et al. 2023; Cannaert et al. 2020).

In July 2022, China implemented a class-wide ban on SC, which led to the development of ADB-FUBIATA as a modified SC to circumvent the regulation. This drug has been seized in drug raids across China, Russia, the United States, Denmark, and Belgium. Predominantly, this drug shows a high selectivity for CB1 receptors with 141% efficacy relative to CP55,940 and very low CB2 receptor affinity (Deventer et al. 2022; Pasin et al. 2022). High CB1 receptor affinity of ADB-4en-PINACA and ADB-FUBIATA has been linked to headache and chest pain, with autopsy findings revealing brain edema and internal bleeding (Simon et al. 2023). These SC compounds mimicked Δ^9^-THC’s psychoactive effects with greater potency and led to cardiac arrhythmias by significantly blocking the hERG channel (Hancox et al. 2023).

In 2018, 5Cl-AKB-48 was detected during a forensic analysis in Kuwait. It was often found mixed with other SC, such as 5F-AKB-48 and 5F-ADB, as well as more commonly seen drugs like methamphetamine, tramadol, and Δ^9^-THC, which highlights the trends of polysubstance abuse patterns (Al-Matrouk et al. 2019). Compounds like FUB-144 and FUB-AKB-48 were frequently found in ‘Spice’ mixtures, with potent action on CB2 receptors. These compounds exhibited 16–20 times greater potency at CB2 over CB1 receptors (Marusich et al. 2022; Hess et al. 2016). However, in a drug discrimination assay in mice, nine novel compounds substituted for Δ^9^-THC with high, moderate, and low potencies (Marusich et al. 2022). 5F-MDMB-PICA, 5F-EDMB-PINACA, MDMB-4en-PINACA had bulky head groups with fluorinated tails that could have contributed to their CB1 selectivity and CNS penetration, while compounds such as MMB-FUBICA, FUB-AKB48, MMB-4en-PICA, FUB-144, or APP-BINACS that lacked the fluorinated tails, minimal substitution in the amide linker group, and or the bulky head reducing their potency from the previous highly potent group of compound but with fluorobenzyl linker that contributed to moderate affinity to CB1 receptor (Marusich et al. 2022).

MDA-19 represents an oxizid group of SC that selectively targets the CB2 receptor and has been widely studied as a promising candidate for therapeutic applications. These include targeted therapy for hepatocarcinoma, and to alleviate neuropathic pain and inflammation without psychoactive effects (Rao et al. 2019; Patel et al. 2024).

The present study examined the behavioral pharmacological effects and potential abuse liability of these eleven compounds. Little or no behavioral testing has been conducted with these compounds, so tests of locomotor activity were conducted using multiple doses to identify the active time course and dose ranges of these compounds. To provide data on the whether the test compounds produce subjective effects similar to Δ^9^-THC, they were tested in rats trained to discriminate Δ^9^-THC. The drug discrimination assay is a well-validated animal model of the subjective effects of behaviorally-active compounds (Horton et al. 2013). The data obtained from these studies can be used to understand the pharmacological activity and potential abuse liability of newer synthetic SCs and provide guidance for scheduling.

## Methods

### Subjects

Male Swiss-Webster mice (*n* = 576) and Sprague–Dawley (*n* = 43) rats were purchased from Envigo (Indianapolis, IN). The mice were approximately 8 weeks old and tested at around 10 weeks of age. Mice were housed in groups of 3–4 on a 12:12-h light/dark cycle (lights on at 7:00 AM) and had free access to food and water except during test sessions. The rats were approximately 90 days old at purchase, were housed individually and maintained on a 12:12-h light/dark cycle. Body weights were kept between 320–350 g by limiting food intake to about 15 g per day, including the food pellets received during operant sessions. Water was always available in the home cage. These protocols adhered to the guidelines for the care and use of laboratory animals and were approved by the University of North Texas Health Science Center Institutional Animal Care and Use Committee.

### Drugs

Delta(9)-tetrahydrocannabinol (Δ^9^-THC), N-[(2S)−1-amino-3,3-dimethyl-1-oxobutan-2-yl]−1-butylindazole-3-carboxamide (ADB-BUTINACA), N-(1-adamantyl)−1-[(4-fluorophenyl)methyl]indazole-3-carboxamide (FUB-AKB-48), N-[(2S)−1-amino-3,3-dimethyl-1-oxobutan-2-yl]−1-pent-4-enylindazole-3-carboxamide (ADB-4en-PINACA), methyl (2S)−2-[[1-(4-fluorobutyl)indole-3-carbonyl]amino]−3,3-dimethylbutanoate (4F-MDMB-BICA), N-(1-amino-3,3-dimethyl-1-oxobutan-2-yl)−1-hexylindazole-3-carboxamide (ADB-HEXINACA), ethyl (2S)−2-[[1-(5-fluoropentyl)indole-3-carbonyl]amino]−3-methylbutanoate (5F-EMB-PICA), N-(1-adamantyl)−1-(4-fluorobutyl)indazole-3-carboxamide (4F-ABUTINACA), [1-[(4-fluorophenyl)methyl]indol-3-yl]-(2,2,3,3-tetramethylcyclopropyl)methanone (FUB-144), N-(1-adamantyl)−1-(5-chloropentyl)indazole-3-carboxamide (5-Cl-AKB-48), (2S)−2-[[2-[1-[(4-fluorophenyl)methyl]indol-3-yl]acetyl]amino]−3,3-dimethylbutanamide (ADB-FUBIATA), and N-(1-hexyl-2-hydroxyindol-3-yl)iminobenzamide (MDA-19) were supplied by the National Institute of Drug Abuse Supply Program. All drugs were dissolved in ethanol/Cremophor EL/0.9% saline (ECS 1:1:18) and administered intraperitoneally at a volume of 10 ml/kg in mice and 1 ml/kg in rats. The solutions were within a pH of 5.5 and 7.5 and were not buffered.

### Locomotor activity

Thirty-two Digiscan locomotor activity testing chambers (model RXYZCM, Omnitech Electronics, Columbus, OH;40.5 × 40.5 × 30.5 cm) were housed within sound-attenuating enclosures. Each enclosure featured a panel of 16 infrared beams with corresponding photodetectors arranged horizontally along the sides of each activity chamber. An incandescent light (23 lx) above each chamber provided dim illumination, and fans maintained an ambient noise level of 80 dB within the chamber. Separate groups of 8 mice were administered the test compounds via intraperitoneal injections with either vehicle (ECS [1:1:18]) or doses of Δ^9^-THC (1, 2.5, 5, 10 or 25 mg/kg), ADB-BUTINACA (0.025, 0.05, 0.1, 0.25, 0.5 or 1 mg/kg), ADB-4en-PINACA (0.1, 0.25, 0.5 or 1 mg/kg), FUB-AKB-48 (0.1, 0.3, 1, 3 or 10 mg/kg), 4F-MDMB-BICA (0.025, 0.05, 0.1, 0.25, 5 or 1 mg/kg), ADB-HEXINACA (0.01, 0.05, 0.1, 0.25, 0.5 or 1 mg/kg), 5F-EMB-PICA (0.1, 0.25, 0.5 or 1 mg/kg), 4F-ABUTINACA (0.1, 0.25, 0.5, 1, 2.5, 5 or 10 mg/kg), FUB-144 (0.1, 0.3, 1, 3 or 10 mg/kg), 5-Cl-AKB-48 (1, 2.5, 5, 10, 25 or 50 mg/kg) or ADB-FUBIATA (1, 2.5, 5, 10, 25, 50 or 100 mg/kg). Each compound was tested with a separate vehicle control group. After the injection, each mouse was placed in the locomotor activity testing apparatus. Horizontal activity, measured by photocell beam interruption, was recorded over a 4- to 6-h period in 10-min intervals. The cannabinoids were tested for Δ^9^-THC-like depressant effects. Testing of the cannabinoids started at 1 mg/kg and continued for at least four doses and the depressant effect was no longer decreasing, up to a maximum of 100 mg/kg.

### Discrimination procedures

Standard behavior-testing chambers (Coulbourn Instruments, Allentown, PA) were connected to IBM-PC compatible computers via interfaces (Med Associates, East Fairfield, VT). The computers were programmed in Med-PC for Windows, version IV (Med Associates, East Fairfield, VT) to operate the chambers and collect data. Rats were trained to discriminate Δ^9^-THC (3 mg/kg) from vehicle (ethanol: Cremophor: saline (1:1:18)) using a two-lever choice method. Rats received injections of either the vehicle or the test compound and were placed in the behavior-testing chambers, where 45 mg food pellets (Bio-Serve, Frenchtown, NJ) served as a reinforcer. One lever was associated with vehicle-injections and the other was associated with Δ^9^-THC injections. A pellet was delivered each time the rat made 10 responses on the lever associated with the injection. The pretreatment time was 30 min for Δ^9^-THC and its vehicle. Training and test sessions ended after rats earned 20 food pellets or after 20 min, whichever occurred first. Rats completed at least 60 training sessions before they were used to test compound substitution. Rats were eligible for testing once they achieved 9 out of 10 sessions at 85% injection-appropriate responding for the first reinforcer and the session total. Training sessions were held on separate days in a double alternating pattern (drug-drug-vehicle-vehicle-drug, etc.) until the training phase was completed. Once substitution testing was introduced, the schedule ensured that at least one saline and one drug test were conducted between each test session (drug-vehicle-test-vehicle-drug-test-drug, etc.). Substitution tests only occurred if rats had shown 85% injection-appropriate responding on the two preceding training sessions. Unlike during training, on test days, both levers were active and would reward the animal on a fixed-ratio 10 schedule for either lever.

Each compound was tested in six rats, with each rat receiving all doses. Vehicle and Δ^9^-THC controls were tested prior to the start of the compound evaluation. Doses and pretreatment times included: ADB-BUTINACA (0.01–0.1 mg/kg; 15 min), FUB-AKB-48 (0.1–1 mg/kg; 60 min), 4D-MDMB-BICA (0.01–0.1 mg/kg; 15 min), 5F-EMB-PICA (0.05–0.5 mg/kg; 15 min), FUB-144 (0.1–5 mg/kg; 30 min), 5-Cl-AKB-48 (1–5 mg/kg; 30 min), ADB-HEXINACA (0.025–0.25 mg/kg; 20 min), ADB-4en-PINACA (0.01–0.25 mg/kg; 15 min), ADB-FUBIATA (10–100 mg/kg; 35 min), 4F-ABUTINACA (0.05–1 mg/kg; 15 min), and MDA-19 (5–100 mg/kg; 30 min). Each compound was tested across doses, from no effect (< 20% drug-appropriate responding) to full effect (≥ 80% drug-appropriate responding), rate suppression (< 20% of the vehicle control), or adverse effects (loss of righting, tremors, convulsions, lethality, etc.). All pretreatment and starting doses were based on locomotor activity assay testing.

### Data analysis

Locomotor activity data were expressed as the mean number of photocell counts in the horizontal plane (ambulation counts) recorded in 10-min intervals. For the dose–response analysis of the ED_50_ calculation, a 30-min period was used, beginning when maximal locomotor suppression was first observed as a function of dose. Origin (OriginLab Corporation, Northampton, MA) was used to estimate the maximal depression caused by each cannabinoid. The ED_50_ values were determined by identifying the dose that produced 50% of the maximal depression from the descending linear portion of the dose–response curve. A two-way ANOVA was performed on horizontal activity counts per 10-min interval, with dose as a between-groups factor and time as a within-group factor. A one-way ANOVA was then conducted on activity counts during the 30-min maximal effects, followed by planned comparisons for each dose versus vehicle control using single-degree-of-freedom F tests.

Drug discrimination data were reported as the mean percentage (± standard error) of drug-appropriate responses during each test session. The response rate was calculated by dividing the total number of responses for each rat by the session time. The response rate data is presented as the average (± standard error) of all rats tested at that dose. Because response suppression might compromise stimulus control, rats that failed to make at least ten responses during a test session were excluded from the analysis of percent drug-appropriate responding for that dose. If three or more rats did not complete the first reinforcer at a given dose, data for that dose were not reported. Graphs of percentage drug-appropriate responding and response rate were plotted as functions of the dose of the test compound on a log scale. Percent drug-appropriate responding was shown only if at least three rats completed the first fixed ratio, while all rats were included in the response rate data. Full substitution was defined as ≥ 80% drug-appropriate responding and not statistically different from the training drug. The potencies of ADB-BUTINACA, FUB-AKB-48, 4F-MDMB-BICA, 5F-EMB-PICA, FUB-144, 5-Cl-AKB-48, ADB-HEXINACA, ADB-4en-PINACA, ADB-FUBIATA, 4F-ABUTINACA, and MDA-19 were calculated by fitting straight lines to the dose–response data for each compound using Origin. These lines were fitted to the linear portion of the dose–effect curves, including no more than one dose producing under 20% of the maximal effect and no more than one dose producing over 80% of the maximal effect. Other doses were excluded from the analysis. Response-rate data were analyzed with one-way repeated-measures ANOVA. Individual effects of doses were compared to vehicle controls using a priori contrasts. Statistical significance was set at *p* < 0.05.

## Results

### Locomotor activity

**Fig. 2:**
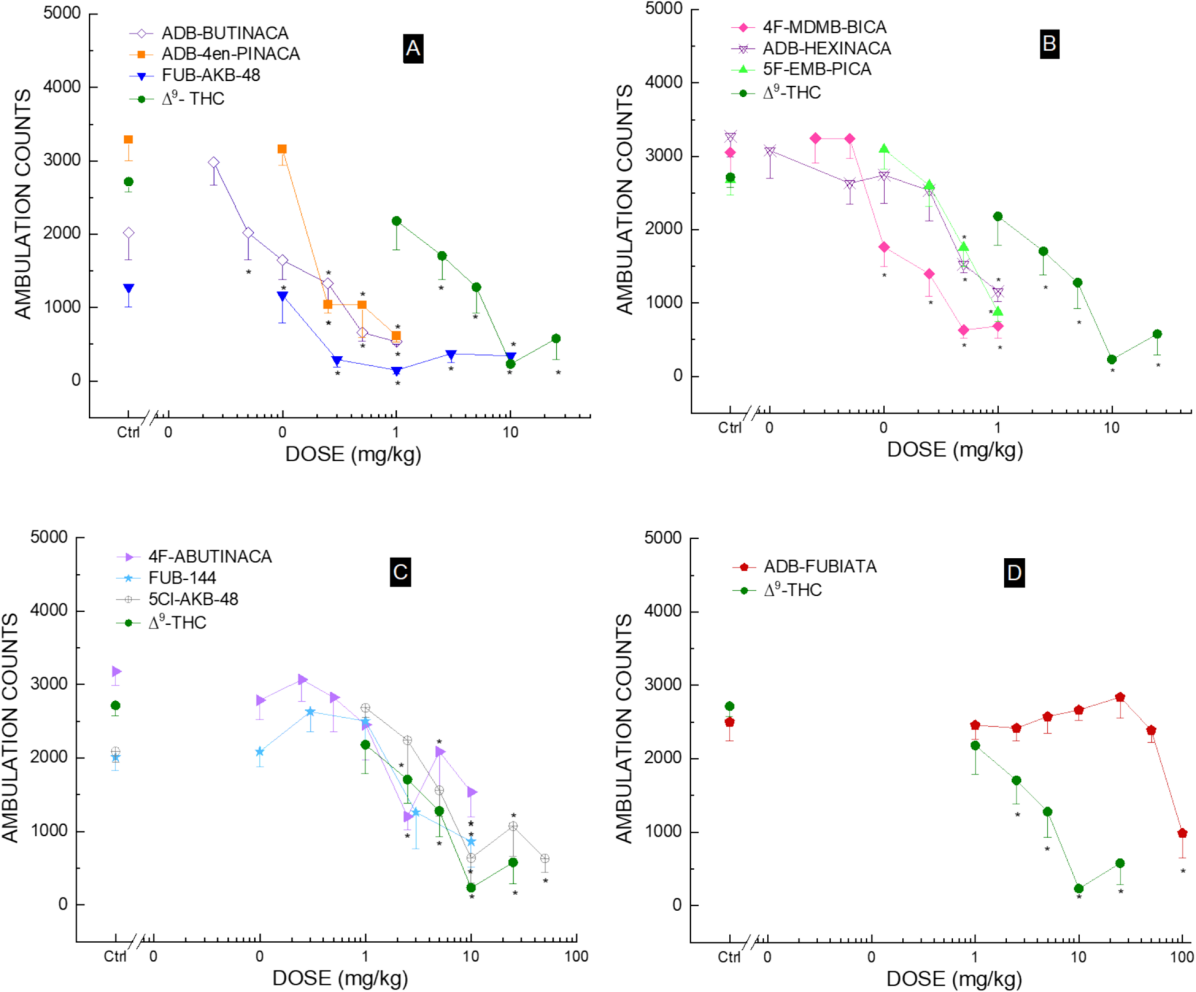
Locomotor activity dose–effect (panels **A**, **B**, **C**, and **D**). Average horizontal activity counts/10 min (± SEM) during the 30 min of peak effect as a function of dose for each of the test compounds. All of the compounds decreased ambulation. *n* = 8 for each dose. Veh indicates vehicle control. * indicates (*p* < 0.05) against vehicle control from one-way repeated-measures ANOVA

Each of the synthetic cannabinoids depressed locomotor activity, similarly to Δ^9^-THC, with similar onset and time course (data not shown). The compounds differed in potency (Table 1), with most compounds being more potent than Δ^9^-THC, except for ADB-FUBIATA. Figure 2 shows the dose response during the earliest 30-min period of maximal effect for each of the test compounds.

Within 30–60 min after injection, the cannabinoid Δ^9^-THC caused dose-dependent locomotor activity that lasted up to 90–120 min following a 10 mg/kg dose [Treatment *F*(5,42) = 10.98, *p* < 0.001, 10-Minute Periods *F*(23,966) = 48.89, *p* < 0.001, Periods x Treatment *F*(115,966) = 1.51, *p* < 0.001]. The period 30–60 min was selected for analysis of dose–response data because this was the time period in which Δ^9^-THC produced maximal effects. Significant depressant effects were observed 30 to 60 min following 2.5, 5, 10 and 25 mg/kg [*F*(5,42) = 10.98, *p* < 0.001].

Treatment with ADB-BUTINACA caused a time- and dose-dependent decrease in locomotor activity. Depressant effects appeared within 10 min of injection and lasted between 30 and 70 min at doses ranging from 0.05 to 0.5 mg/kg [Treatment F (6,49) = 2.20, *p* = 0.058, but there was a significant effect for 10-min; Periods F (47,2303) = 16.84, *p* < 0.001]; Periods x Treatment F (282,2303) = 2.51, *p* < 0.001]. At the period of maximal effect (0–30 min), there was a significant locomotor depression at doses of 0.05, 0.1, 0.25, 0.5, and 1 mg/kg [F (6,49) = 18.17, *p* < 0.001].

Administration of ADB-4en-PINACA caused a time- and dose-dependent decrease in locomotor activity. Depressant effects appeared within 10 min after a 0.25 mg/kg injection and lasted for 50 min [Treatment F(4,35) = 4.61, *p* < 0.001, 10-Minute Periods F(23,805) = 17.64, *p* < 0.001, and a significant interaction of Periods x Treatment F(92, 805) = 3.65, *p* < 0.001]. Significant depressant effects were observed within the 0 to 30-min interval for doses of 0.25, 0.5, and 1 mg/kg [F(4,35) = 23.91, *p* < 0.001]. Convulsions were observed in 1 of 8 mice following 1 mg/kg ADB-4en-PINACA.

FUB-AKB-48 caused a dose- and time-dependent reduction in locomotor activity. Depressant effects appeared within 40 min after injection and lasted for 120 min at 0.3 mg/kg [Treatment F(5,42) = 3.87, *p* = 0.006, F(47,1974) = 26.48, *p* < 0.001; Periods x Treatment F(235,1974) = 1.54, *p* < 0.001]. The peak effect analysis was conducted at 60–90-min intervals, indicating a significant effect for the 0.3, 1, 3, and 10 mg/kg doses [*F*(5,42) = 5.77, *p* < 0.001].

Administration of 4F-MDMB-BICA caused a time- and dose-dependent decrease in locomotor activity. Depressant effects began within 10 min after injection and lasted from 50 to 90 min at doses of 0.1 to 0.5 mg/kg [Treatment F(6,49) = 3.52, *p* = 0.006; 10-Minute Periods F(47,2303) = 18.24, *p* < 0.001; Periods x Treatment F(282,2303) = 2.22, *p* < 0.001]. During the time of maximal effect (0–30 min) depressant effects occurred following 0.1, 0.25, 0.5, and 1 mg/kg. [F(6,49) = 17.49, *p* < 0.001].

ADB-HEXINACA exhibited a dose- and time-dependent reduction in locomotor activity at doses of 0.5–1 mg/kg. The effect appeared 10 min after injection of 0.5 and 1 mg/kg and lasted for 70 to 80 min [Treatment F(6,49) = 2.23, *p* = 0.056; 10-Minute Periods F(23,1127) = 51.08, *p* < 0.001; Periods x Treatment *F*(136, 1127) = 2.31, *p* < 0.001]. The 0–30 min time period was analyzed, and a depressant effect was evident at the 0.5 and 1 mg/kg doses [*F*(6,49) = 6.63, *p* < 0.001].

5F-EMB-PICA caused a time- and dose-dependent decrease in locomotor activity. Depressant effects appeared within 10 min after injection and lasted for 30 min at the 0.5 and 1 mg/kg doses [Treatment F(4,35) = 1.22, *p* = 0.320; 10-Minute Periods F(47,1645) = 15.93, *p* < 0.001; Periods x Treatment F(188,1645) = 1.67, *p* < 0.001]. The 0–30 min maximal effect showed depressant effects for the 0.5 and 1 mg/kg doses [Treatment F(4,35) = 13.93, *p* < 0.001].

FUB-144 caused a dose- and time-dependent decrease in locomotor activity, with depressant effects appearing within 20 min of injection and lasting for 40 min at a dose of 10 mg/kg [Treatment F(5,42) = 2.1, *p* = 0.085; 10-Minute Periods F(47,1974) = 18.91, *p* < 0.001]; Periods x Treatment F(235, 1974) = 1.36, *p* < 0.001. Within the 10–40-min time frame, the maximum depressant effect was significant for 10 mg/kg [F(5,42) = 4.86, *p* = 0.001]. A locomotor stimulant effect was evident during the second hour following injection of 0.3 mg/kg FUB-144.

4F-ABUTINACA caused a time- and dose-dependent reduction in locomotor activity. The effect began within 10 min after administering 2.5 mg/kg and lasted for 80 min. [Treatment F(7,56) = 5.39, *p* < 0.001, 10-Minute Periods F(23,1288) = 41.80, *p* < 0.001; Periods x Treatment F(161,1288) = 1.21, *p* = 0.048]. The 0- to 30-min interval showing maximal depressant effect demonstrated significant suppression at doses of 2.5, 5, and 10 mg/kg [F(7,56) = 3.90, *p* = 0.002].

Treatment with 5-Cl-AKB-48 resulted in a time- and dose-dependent decrease in locomotor activity. The depressant effects occurred within 20 min of injection and lasted for 70 min at 10 mg/kg [Treatment F(6,49) = 5.06, *p* < 0.001; 10-Minute Periods F(47,2303) = 32.55, *p* < 0.001; Periods x Treatment F(282,2303) = 1.69, *p* < 0.001]. The 20–50 min period showed a significant depressant effect for doses of 10, 25, and 50 mg/kg [F(6,49) = 6.06, *p* < 0.001].

ADB-FUBIATA caused a time- and dose-dependent decrease in locomotor activity. The depressant effects appeared within 20 min after injection of 100 mg/kg and lasted for 40 min. [Treatment F(7,56) = 0.94, p = 0.484, a significant effect for 10-Minute Periods F(23,1288) = 95.74, *p* < 0.001; Periods x Treatment F(161, 1288) = 1.61, *p* < 0.001]. There was a significant depressant effect in the 10–40-min time period following 100 mg/kg [*F*(7,56) = 6.17, *p* < 0.001].

### Drug discrimination

**Fig. 3:**
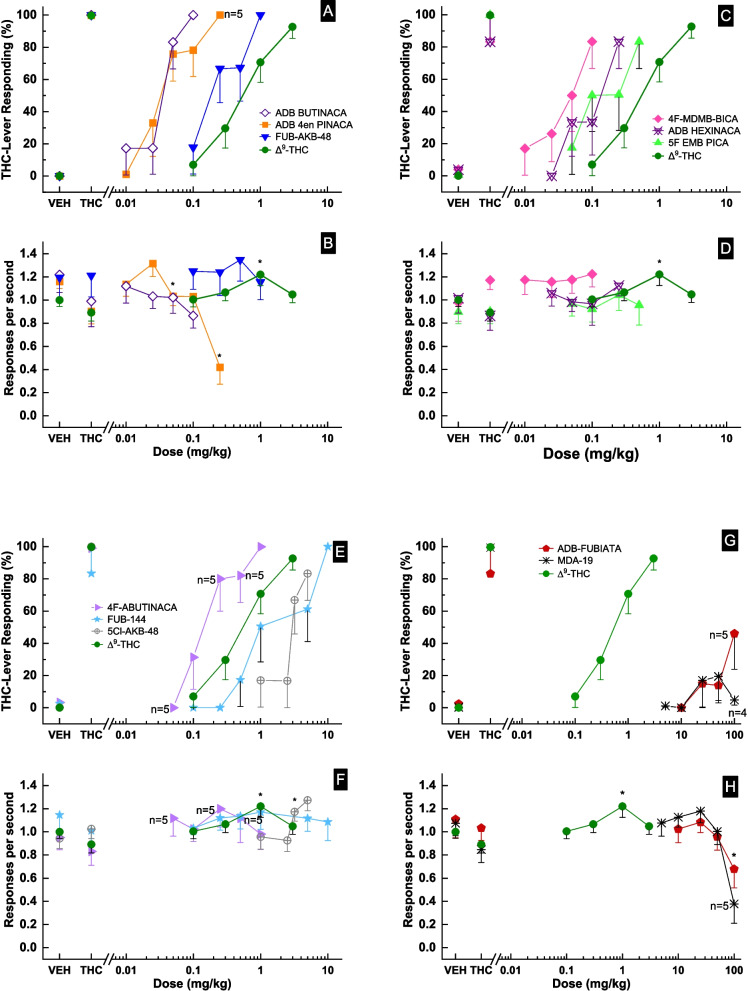
Substitution for the discriminative stimulus effects of Δ^9^-THC (panels **A**—**H**). The top panels show the percentage of total responses made on the drug-appropriate lever. Bottom panels show the rate of responding in responses per second (r/s). *n* = 6 except where shown. Ctrl indicates vehicle and Δ^9^-THC control values. * indicates response rate different from vehicle control (*p* < 0.05) from one-way repeated-measures ANOVA

Δ^9^-THC produced dose-dependent increases in drug-appropriate responding, peaking at 93 ± 7% after a 3 mg/kg training dose (Fig. 3). ADB-BUTINACA, ADB-4en-PINACA, FUB-AKB-48, 4F-MDMB-BICA, ADB-HEXINACA, 5F-EMB-PICA, 4F-ABUTINACA, FUB-144, and 5-Cl-AKB-48 each fully substituted for the discriminative stimulus effects of Δ^9^-THC, producing ≥ 80% drug-appropriate responding (Fig. 3). Potencies of the test compounds are shown in Table 1. ADB-4en-PINACA decreased response rate following 0.05 and 0.25 mg/kg *F*(5,25) = 10.26, *p* < 0.001, whereas 5-Cl-AKB-48 increased response rate *F*(4,20) = 3.60, *p* = 0.023. ADB-BUTINACA *F*(4,20) = 1.14, *p* = 0.368, FUB-AKB48 *F*(4,20) = 0.63, *p* = 0.650, 4F-MDMB-BICA *F*(4,20) = 0.05, *p* = 0.517, ADB-HEXINACA *F*(4,20) = 0.56, *p* = 0.696, 5F-EMB-PICA (EMB 2201) *F*(4,20) = 0.43, *p* = 0.788, 4F-ABUTINACA *F*(5,20) = 0.80, *p* = 0.565, and FUB-144 *F*(6,30) = 0.37, *p* = 0.891 failed to produce a significant effect on response rate.

Conversely, ADB-FUBIATA produced only 46 ± 22% Δ^9^-THC-appropriate responding at 100 mg/kg, with the response rate decreasing to 61% of the vehicle control *F*(4,20) = 3.01, *p* = 0.040. MDA-19 did not substitute (< 20% drug-appropriate responding) for the discriminative effects of Δ^9^-THC up to doses that decreased responding *F*(5,20) = 6.41, *p* = 0.001. Only five of the six rats were tested at 100 mg/kg.

## Discussion

The current study compared the locomotor activity (LMA) and drug discrimination (DD) effects of eleven novel synthetic cannabinoids (SC) with those of ∆^9^-THC in rodents. The data are consistent with previous findings on cannabinoids, showing strong Δ^9^-THC-like effects. Most of the SC were more potent than ∆^9^-THC (summarized in Table 1). In the LMA assay, most tested compounds reached peak depressant effects at low doses within 10–40 min after injection. ED_50_s ranged from 0.12 mg/kg to 87.7 mg/kg. ADB-BUTINACA was the most potent, while ADB-FUBIATA was the least potent, producing depressant effects only at 100 mg/kg. Additionally, mice injected with 1 mg/kg ADB-4en-PINACA experienced convulsions within 30 min. The rank order of potency in the locomotor activity assay was ADB-BUTINACA = ADB-4en-PINACA = FUB-AKB-48 = 4F-MDMB-BICA > ADB-HEXINACA > 5F-EMP-PICA > 4F-ABUTINACA > THC > FUB-144 > 5Cl-AKB-48 > ADB-FUBIATA (Table 1).

In the drug discrimination test, all SC except ADB-FUBIATA and MDA-19 fully substituted for Δ^9^-THC, exhibiting a similar potency ranking to that for locomotor depression. ADB-BUTINCA, ADB-4en-PINACA, and 4F-MDMB-BICA were the most potent, and 5-Cl-AKB-48 was the least potent of the compounds that substituted for ∆^9^-THC. The DD ED_50_s for SC that fully substituted were generally 2- to 14-fold lower than the LMA ED_50_s, indicating, in general, that smaller doses were needed to produce interoceptive effects than to suppress locomotor activity. However, this was not the case for FUB-AKB-48 that had equivalent potency for locomotor depression and THC substitution. Also, ADB-4en-PINACA substituted for ∆^9^-THC at doses that suppressed response rate, even though fivefold higher doses were needed to suppress unmotivated locomotor activity. MDA-19 produced little or no effect in the drug discrimination assay, and motor activity data were not available. Therefore, the rank order of potency for DD can be summarized as ADB-BUTINACA = ADB-4en-PINACA = 4F-MDMB-BICA > ADB-BUTINACA = 4F-ABUTINACA = 5F-EMP-PICA > FUB-AKB-48 > THC > FUB-144 > 5Cl-AKB-48. ADB-FUBIATA and MDA-19 produced little or no THC-like responding when tested at much higher doses (10–100 mg/kg) than the other SC.

Synthetic cannabinoid structures typically include regions such as the core, linker-head, and tail groups that can be easily modified to change activity (Alves et al. 2020; Potts et al., 2020). Indazole-3-carboxamides such as ADB-BUTINACA, FUB-AKB-48, ADB-HEXINACA, and ADB-4en-PINACA have a strong affinity for the CB1 receptor due to their hydrophobic side chains and hydrogen-bonding carboxamide groups (Kronstrand et al. 2022; Deventer et al. 2022; Sparkes et al. 2022a, 2024). This structure allows them to act as full agonists with very high potency at the CB1 receptor, which is reflected in both locomotor and drug discrimination assays, with ED_50_s < 0.55 (Table 1). These compounds also had a faster onset of action in the present study, occurring within 10 min and lasting about 80 min. These results are quite similar to those reported for ADB-4en-PINACA, in which locomotor activity was suppressed in the same dose range as in the present study (Qiao et al. 2025). In that study, hypothermia, analgesia, and catalepsy were also observed in the same dose range.

4F-MDMB-BICA, 5F-EMB-PICA, and 4F-ABUTINACA also had relatively higher potencies in both behavioral assays (ED_50_s < 1.9) than FUB-144, 5Cl-AKB-48, and ADB-FUBIATA (Table 1). These compounds have fluoride substitutions that are known to increase lipophilicity, enhance BBB permeability, and improve CB1 receptor binding (Gillis et al. 2015; Canazza et al. 2016). It is known that SC compounds with an indole core have weaker binding compared to SCs with an indazole core (Schoeder et al. 2018). However, in our studies, 4F-MDMB-BICA was found to be significantly more potent in the DD assay, with an ED_50_ of 0.041, compared to the other two fluorinated SCs (FUB-AKB-48 and 5 F EMB PICA; ED_50_’s > 0.16, and it was equipotent to some of the highly potent ADB compounds in producing interoceptive effects; ED_50_’s < 0.038 (Table 1). Both 4F-MDMB-BICA and 5F-EMB-PICA have an indole core and are expected to have similar affinities for the CB1 receptor. However, both in the literature and our studies show that 4F-MDMB-BICA was more potent at the CB1 receptor than 5F-EMB-PICA (Cannaert et al. 2020; Dvorácskó et al. 2023). The weak affinity to the CB1 receptor by 5F-EMB-PICA could be attributed to the amide head and 5-fluropentyl tail, which is less effective in binding at the receptor (Sparkes et al. 2022b; Dea 2023). In contrast, 4F-BUTINACA, despite having an indazole core, was less potent than both 4F-MDMB-BICA and 5F-EMB-PICA in LMA, possibly due to the amide versus the ester linker group (Hess et al. 2016; Green et al. 2025).

5Cl-AKB-48 showed a lower potency with ED_50_ = 6.11 in LMA and ED_50_ = 3.21 in DD, whereas FUB-AKB-48 exhibited higher potency with ED_50_ = 0.19 in LMA and ED_50_ = 0.23 in DD assays. These two compounds are structurally similar except for the tail group: a 5-chloropentyl chain in 5Cl-AKB-48 and a 4-fluorobenzyl chain in FUB-AKB-48, and this structural difference explains the fat solubility and lipophilic nature of FUB-AKB-48, as well as its better CNS penetration (Gillis et al. 2015; Canazza et al. 2016; Shi et al. 2025). Furthermore, the chloropentyl group in 5Cl-AKB-48 most likely led to a faster onset, shorter duration, and less stable CB1 binding compared to FUB-AKB-48, as evidenced by the depressant effect in LMA, which lasted only 120 min. In contrast, another similar compound to FUB-AKB-48, FUB-144, exhibited moderate potency in both behavioral assays (LMA ED_50_ = 6.1 and DD ED_50_ = 1.44). FUB-AKB-48 showed greater CB1 selectivity, while FUB-144 was 23 times more selective for the CB2 receptor. This difference in potency can be attributed to the receptor affinities for CB1 and CB2 receptors. The tetramethyl cyclopropyl bulky head group in FUB-144 contributed to its selectivity and enhanced binding to the CB2 receptor (Hess et al. 2016; Thomas et al. 2017).

The ineffectiveness of ADB-FUBIATA in both assays is likely due to its reduced interaction with CB1 receptors, which is caused by its bulky structure. Although this compound is also indole-based, it acts as a depressant only at very high doses in motor activity and produced only 46% ∆^9^-THC-like responding in the drug discrimination assay (Table 1). Similarly, ADB-FUBIATA has low selectivity for the CB1 receptor, shows 43% lower efficacy compared to JWH-18, and has negligible activity at CB2 receptors. The addition of a methylene group to the acetamide linker group of ADB-FUBICA may influence its interaction with the CB1 receptor and could explain its failure to substitute in the DD assay, along with reduced in vitro potency when compared to other indole-based compounds at the CB1 receptor (Deventer et al. 2022; Watanabe et al. 2023; Banister et al. 2015).

MDA-19 (BZO-HEXOXIZID) was initially developed as a CB2-selective agonist, with this selectivity primarily attributed to its benzoyl moiety; however, the addition of a hexyl tail resulted in decreased CB1 activity (Deventer et al. 2022; Diaz et al. 2008; Xu et al. 2010). In the current study, MDA-19 failed to substitute for Δ^9^-THC in rat DD. Another study reported that in mice, MDA-19 only partially substituted for Δ^9^-THC at 30 mg/kg in DD, and at higher doses (50–100 mg/kg), there was a significant decrease in the percentage of responding on the THC-associated aperture, which also resulted in reductions of response rate compared to the vehicle (Patel et al. 2024). In a published study, MDA-19 showed strong antiallodynic effects in the rat model of neuropathic pain; however, no significant change in exploratory behavior was observed when tested in an open field. The investigators further concluded that MDA-19 acts peripherally, and not centrally, making it effective in a pain model without producing any central effects that influence motor activity (Diaz et al. 2008). This could also explain why, in the current study, it did not substitute for Δ^9^-THC in the rat DD task.

## Conclusions

In summary, the evaluation of eleven synthetic cannabinoids in behavioral pharmacological assays reveals that structural differences and affinity of each compound for CB1 and CB2 receptors influenced their behavioral effects. As previously discussed, the core, linker head, and tail regions of the structure affect binding to cannabinoid receptors and BBB penetration. The more potent compounds feature an indazole core, a tert-leucinate ester head group, and highly lipophilic tail groups, such as isobutyl and hexyl, which seem to confer a high affinity for CB1 receptors and fully substitute for the subjective effects of Δ^9^-THC. In contrast, moderate potency compounds have a linker head group with a methyl ester, resulting in better binding to CB1 receptors than a carboxamide or adamantyl group. Additionally, halogenated aryl tails, such as 4-fluorobenzyl, show a better affinity and binding to CB1 receptors than fluorinated alkyl chains. Furthermore, fluorine aryl tails exhibit better BBB penetration than their chlorinated and brominated counterparts. Linker groups composed of methylene and acetamide show poor conformational stability when interacting with CB1 receptors, resulting in less stable receptor interactions. While the benzimidazole ring favors CB2 receptors and is less active at CB1 receptors, it is therefore less psychoactive compared to SCs with an indole or indazole core ring.

The wide range of potencies in the behavioral assessments across the tested compounds reflects their differential affinity to the CB1 receptor. As previously described, high affinity to CB1 receptors results in clinical outcomes such as CNS depression, agitation, anxiety, seizures, psychosis, and cardiovascular effects (Kavanagh et al. 2022; Police News and Information Related 4F-MDMB-BICA n.d.; Simon et al. 2023; Hancox et al. 2023). Autopsy findings further indicate that exposure to SC can contribute to brain edema and internal bleeding (Simon et al. 2023). Collectively, these findings suggest that highly potent SC could drive potential toxicities in humans. Most of the compounds tested fully substituted for the discriminative stimulus effects of Δ^9^-THC and likely have abuse liability comparable to other synthetic cannabinoids. Ideally, such conclusions would be buttressed with self-administration and/or conditioned place preference studies, but since cannabinoids are not consistently self-administered and are more likely to produce place aversion, drug discrimination remains the best alternative for assessing abuse liability e.g., (Diaz et al. 2008). This study emphasizes the critical role of behavioral pharmacology outcomes that can be used to determine the potency of synthetic SC, as the outcomes mirror the structure–activity relationships reported in the literature. Therefore, behavioral pharmacological testing can provide an early detection of the abuse liability and toxicity of emerging SCs. These findings need to be confirmed with in vitro pharmacology.

## Data Availability

All data are the property of the National Institute on Drug Abuse Addiction Treatment Discovery Program. Interested parties may contact the Program for technical reports and raw data.

## Abbreviations

SC: Synthetic cannabinoids
∆9-THC: Delta-9-tetrahydrocannabinol
LMA: Locomotor activity
DD: Drug discrimination
DEA: Drug Enforcement Administration
EMCDDA: European Monitoring Center for Drugs and Drug Addiction
ADB-BUTINACA: N-[(2S)-1-amino-3,3-dimethyl-1-oxobutan-2-yl]-1-butylindazole-3-carboxamide
FUB-AKB-48: N-(1-adamantyl)-1-[(4-fluorophenyl)methyl]indazole-3-carboxamide
ADB-4en-PINACA: N-[(2S)-1-amino-3,3-dimethyl-1-oxobutan-2-yl]-1-pent-4-enylindazole-3-carboxamide
4F-MDMB-BICA: Methyl (2S)-2-[[1-(4-fluorobutyl)indole-3-carbonyl]amino]-3,3-dimethylbutanoate
ADB-HEXINACA: N-(1-amino-3,3-dimethyl-1-oxobutan-2-yl)-1-hexylindazole-3-carboxamide
5F-EMB-PICA: Ethyl (2S)-2-[[1-(5-fluoropentyl)indole-3-carbonyl]amino]-3-methylbutanoate
4F-ABUTINACA: N-(1-adamantyl)-1-(4-fluorobutyl)indazole-3-carboxamide
FUB-144: [1-[(4-Fluorophenyl)methyl]indol-3-yl]-(2,2,3,3-tetramethylcyclopropyl)methanone
5-Cl-AKB-48: N-(1-adamantyl)-1-(5-chloropentyl)indazole-3-carboxamide
ADB-FUBIATA: (2S)-2-[[2-[1-[(4-fluorophenyl)methyl]indol-3-yl]acetyl]amino]-3,3-dimethylbutanamide
MDA-19: N-(1-hexyl-2-hydroxyindol-3-yl)iminobenzamide
